# Biphasic cartilage repair implant versus microfracture in the treatment of focal chondral and osteochondral lesions of the knee: a prospective, multi-center, randomized clinical trial

**DOI:** 10.1186/s10195-024-00802-1

**Published:** 2024-11-30

**Authors:** Tzu-Hao Tseng, Chao-Ping Chen, Ching-Chuan Jiang, Pei-Wei Weng, Yi-Sheng Chan, Horng-Chaung Hsu, Hongsen Chiang

**Affiliations:** 1 Department of Orthopaedic Surgery, National Taiwan University Hospital, 7 Chungsan South Road, Taipei City, 10002 Taiwan; 2 Department of Orthopaedics, Taichung Veterans General Hospital, Taichung City, Taiwan; 3 Department of Health Services Administration, China Medical University, Taichung City, Taiwan; 4 Department of Acupressure Technology, Jen-Teh Junior College of Medicine, Nursing and Management, Miaoli, Taiwan; 5 Department of Orthopaedic Surgery, Fu Jen Catholic University Hospital, New Taipei City, Taiwan; 6 Department of Orthopaedic Surgery, Taipei Medical University-Shuang Ho Hospital, Ministry of Health and Welfare, New Taipei City, Taiwan; 7 Department of Orthopaedic Surgery, Chang Gung Memorial Hospital-Linkou Branch, Taoyuan City, Taiwan; 8 Department of Orthopaedic Surgery, China Medical University Hospital, Taichung City, Taiwan; 9 Department of Biomedical Engineering, National Taiwan University Hospital, Taipei City, Taiwan

**Keywords:** Cartilage, Osteochondral lesion, Biphasic cartilage-repair implant, Osteoarthritis, Microfracture

## Abstract

**Background:**

Autologous minced cartilage is a method for cartilage defect repair, and our study focuses on a newly developed biphasic cylindrical osteochondral construct designed for use in human knees. We aimed to compare its clinical effectiveness and safety with microfracture, the commonly utilized reparative treatment for knee chondral or osteochondral defects.

**Materials and methods:**

Conducted as a prospective multicenter, randomized controlled, non-inferiority trial across nine hospitals, the study involved 92 patients with International Cartilage Repair Society (ICRS) grade 3 to 4 chondral or osteochondral lesions on femoral condyles. Patients were evenly randomized to receive either the biphasic cartilage-repair implant (BiCRI) or microfracture. Functional outcomes and safety assessments were conducted at postoperative intervals of 6 weeks and 3, 6, and 12 months. Primary and secondary endpoints included International Knee Documentation Committee (IKDC) 2000 Subjective Knee Evaluation Form score improvement, the grade distribution in the IKDC 2000 Knee Examination Form, and various assessments, such as the Knee Injury and Osteoarthritis Outcome Score (KOOS), visual analog scales (VASs) for pain, MRI findings, and arthroscopic findings at 12 months.

**Results:**

Out of the initial participants, 47 in the BiCRI group and 45 in the microfracture group completed the follow-up. At 12 months, the mean change in IKDC total score was 25.56 ± 18.48 for BiCRI and 27.51 ± 23.65 for microfracture. The 95% confidence interval (CI) for the score difference (BiCRI minus microfracture) was − 6.95, exceeding the non-inferiority margin of − 12. Secondary endpoints indicated comparable functional outcomes, and arthroscopic findings demonstrated more fully regenerated cartilage in the BiCRI group.

**Conclusion:**

Based on the IKDC 2000 Subjective Knee Evaluation Form score, BiCRI proved non-inferior to microfracture at 12 months. Short-term functional outcomes were comparable to those with microfracture, while arthroscopic findings showed more complete cartilage regeneration in the BiCRI group. Consequently, BiCRI emerges as a viable alternative for treating chondral or osteochondral defects.

***Level of evidence*:**

Level 2, multi-center, randomized clinical trial.

*Trial registration*: Name of the registry: ClinicalTrials.gov. Trial registration number: NCT01477008. Date of registration: 11/14/2011. URL of trial registry record: clinicaltrials.gov/study/NCT01477008

## Introduction

Focal articular cartilage and osteochondral defects in the knee joint are common issues, with full-thickness cartilage involvement observed in more than 10% of patients undergoing knee arthroscopy [1]. These defects often result in knee pain, swelling, and dysfunction, significantly impacting the quality of life [2]. Due to the limited regenerative potential of cartilage, untreated lesions may progress to advanced osteoarthritis (OA) [3]. Conventional palliative or reparative treatment options, including debridement, abrasion chondroplasty, and marrow stimulation such as microfracture, are effective in providing symptom relief [4]. Among these approaches, microfracture involves creating multiple perforations in the subchondral bone, exposing the bone marrow, and subsequently forming a “superclot” in the defect. This process facilitates the recruitment of mesenchymal stem cells for lesion repair [5], making microfracture the most frequently used reparative approach [6]. However, the resulting repaired cartilage is composed of fibrocartilaginous tissue rather than hyaline cartilage, and the wear characteristics of fibrocartilage are inferior to those of hyaline cartilage [7]. To address this limitation, regenerative procedures like autologous cartilage implantation (ACI) have been developed, which aim to restore the articular surface with hyaline-like cartilage [8]. The clinical results from these regenerative procedures are comparable to those of conventional surgeries [9–12].

In earlier models of ACI, cultivated chondrocytes were placed in a defect sealed with a periosteum patch or collagen membrane [13]. These models necessitate delicate surgical techniques and present significant drawbacks, such as an uneven cell distribution and cell leakage. Consequently, matrix-associated autologous chondrocyte implantation (MACI) was developed [14, 15]. Biodegradable materials carrying chondrocytes act as a temporary scaffold, ensuring the cells are maintained at the focal lesion. As it is an easier technique and gives comparable clinical results to ACI, MACI has become a more preferable option. Although MACI has now been recognized as a promising treatment [16–18], and current culture-expansion techniques do not compromise the chondrogenic potential of chondrocytes, MACI remains a two-step procedure. The requirement for a second surgery could discourage patients. Additionally, neither ACI nor MACI alone is suitable for osteochondral lesions, as the cartilage graft is prone to failure without sufficient subchondral support. These concerns limit the clinical application of these regenerative techniques.

In contrast to conventional chondrocyte implantation techniques, the use of minced cartilage represents a practical approach for one-step cartilage regeneration. Unlike isolated chondrocytes, the chondrocytes in minced cartilage remain in their natural environment, preserving their original phenotype more effectively [19–21]. This “optimal-growth condition” reduces the demand for cells in cartilage regeneration, eliminating the need for a culture-expansion process. Building on the principles and concerns associated with MACI, we have developed a biphasic cartilage repair implant (BiCRI) designed for the implantation of “double-minced” autologous cartilage. This construct is loaded with autologous cartilage processed sequentially through mechanical mincing using a power-driven pulverizer and chemical mincing using enzymatic dissociation. It can be implanted into a focal articular cartilage or osteochondral defect in a single-stage seed-and-implant surgery. The 2-year and 5-year outcomes of a clinical feasibility study have been reported [22, 23]. The promising results demonstrate that this biphasic construct is a safe and effective solution for osteochondral defects.

Having established the safety and feasibility of the biphasic osteochondral composite, we proceeded to conduct a multicenter, randomized controlled, non-inferiority trial to assess its clinical outcomes. The null hypothesis for this study posited that the results of chondral or osteochondral defects treated with the biphasic osteochondral composite would be inferior to those treated with microfracture surgery.

## Materials and methods

### Study design

This multicenter, randomized controlled, non-inferiority trial was conducted across nine hospitals in Taiwan following approval by the institutional review board or institutional ethics committee of each participating hospital. The trial was registered on the Clinical Trials Open Registry (http://clinicaltrials.gov, ID: NCT01477008). The protocol and subject-related documents were reviewed and approved by the Taiwan Food and Drug Administration (TFDA). These institutions also approved the 1-year blinding protocol. Microfracture surgery, being the most commonly utilized reparative approach, was selected as the control treatment. In a prior study, patients treated with microfracture surgery exhibited an increase in the International Knee Documentation Committee (IKDC) overall score from a pre-operative value of 41.1 ± 12.3 points to 70.2 ± 14.7 points, representing a 29.1-point improvement [24]. Sparingly, we established the non-inferiority margin at 12 points, approximately 60% of the effect seen with microfracture surgery, with the standard deviation of the IKDC score set at 20 points. Based on these assumptions, the calculated number of patients required to validate the non-inferiority of the investigational group to the control group, with a one-sided statistical significance level of 2.5% and a power of 80%, is 38 patients per group. To account for potential exclusions affecting 20% of the subjects in the final evaluation, we increased the sample size to include 46 patients in each group.

Every patient provided informed consent before participating in the study. Participants were thoroughly informed during the consent process that they would remain blinded to their treatment unless complications at the surgical site required reoperation on the chondral or osteochondral lesion. Even if a patient withdrew from the study for reasons unrelated to the surgical site, their treatment allocation would remain blinded for 1 year post-surgery. A data safety monitoring board (DSMB) comprising a medical doctor, an independent statistician, and a clinical trial expert was established to safeguard the participants' interests, assess intervention safety while maintaining the blinding, and oversee the trial’s conduct and integrity.

Patients with symptomatic chondral or osteochondral defects of the medial condyle, lateral condyle, or the trochlea of the distal femur were invited to participate if they met the following inclusion criteria: (1) age < 55 years with a single lesion diagnosed by arthroscopic examination and magnetic resonance imaging (MRI); (2) a lesion size of less than 23 mm × 12.5 mm; (3) a lesion of International Cartilage Repair Society (ICRS) grade 3–4, Outerbridge grade 4, or osteochondritis dissecans grade 3–4; (4) skeletally mature as determined by plain roentgenography, with closure or absence of the physeal plate at the distal femur and proximal tibia. Patients were excluded if they had other lesions > grade II on the articular surface of the tibia or patella, prior surgical treatment of the target lesion, a lesion requiring bone grafting, rheumatoid arthritis, another inflammatory arthritis, severe meniscal damage (defined as > 50% of the meniscus missing or a radial tear extending to the meniscal–synovial junction), knee stiffness (flexion contracture > 10° or flexion degree < 115°), body mass index (BMI) > 35.0, local or systemic infection (except for an asymptomatic urinary tract infection), pregnancy, or breastfeeding. Once the lesion size was confirmed intraoperatively by an arthroscopic procedure to meet the inclusion criteria, the patients were randomized to receive either BiCRI implantation or microfracture surgery.

### Biphasic cartilage repair implant

**Fig. 1:**
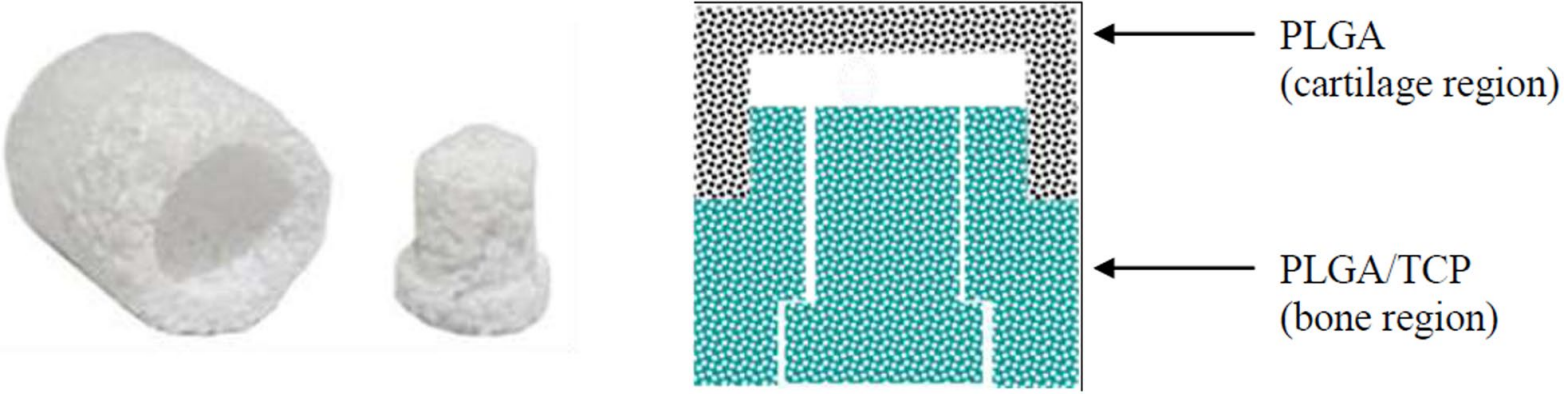
The biphasic osteochondral construct. The design features a barrel-and-plug structure, enabling easy insertion of minced cartilage through the opening on the osseous side. Once the cartilage graft is loaded, the plug is secured to enclose a flat chamber, positioning the graft between the plug and the chondral phase of the construct

The biphasic construct was produced using a modified solvent-merging and particulate-leaching technique, as described previously [25]. This porous cylindrical structure comprised two distinct phases: a chondral phase and an osseous phase. The chondral phase, accounting for one-sixth of the total height, was composed of polylactic-*co*-glycolic acid (PLGA). The remaining portion constituted the osseous phase, made from a composite of PLGA and tricalcium phosphate (TCP) (Fig. 1).

A flat chamber, 6.5 mm in diameter and 1 mm in height, was positioned between the chondral and osseous phases, serving as a reservoir for the double-minced autologous cartilage graft. The final construct measured 8.5 mm in both diameter and height. All constructs used in this study were manufactured in a laboratory adhering to good manufacturing practice (GMP) standards. Preclinical evaluations, including toxicology testing and animal studies, had been conducted [26], and the construct's effectiveness in promoting cartilage regeneration was confirmed in a porcine model [20, 27].

### Surgical procedures and postoperative protocol

All surgical procedures were performed by ten sports-fellowship-trained surgeons, each with over 10 years of experience at their respective hospitals. Prior to the study, these surgeons underwent specialized training on the implant and participated in simulated knee surgery using anatomical models to ensure consistency in surgical technique. Routine knee arthroscopy was performed first to locate the lesions. Depending on the lesion sites, a longitudinal mini-arthrotomy along the medial or lateral border of the patellar tendon was made to approach the defect. For the BiCRI group, the details of the surgical procedures were described previously [22, 23]. In brief, an 8-mm cylindrical hole was created with a cylinder punch to maximally cover the lesion. The cartilage of acceptable quality within the punched area was excised and collected as part of the autograft. Additional autograft was curette harvested from the non-articulating margin of the affected condyle to achieve a total volume of 0.15 cm^3^ cartilage. The cartilage was immersed immediately in sterile saline and morselized with a specially designed tissue pulverizer with a sieve to obtain particles smaller than 1000 μm. The particles were further dissociated with collagenase (Librase, Roche Diagnostics, Mannheim, Germany) at 37 °C for 20 min. After the removal of collagenase through copious rinses with saline, the cartilage graft was transferred to the flat chamber in the BiCRI. The prepared BiCRI was then pressed into the previously punched hole. The patients received either one plug (lesion size ≤ 12.5 mm × 12.5 mm) or two plugs (lesion size ≥ 12.5 mm × 12.5 mm and ≤ 12.5 mm × 23 mm). For the microfracture group, multiple holes were made using 1.5-mm-diameter awl to a depth of 5 mm at distances of 3 to 4 mm.

Postoperative visits were scheduled at 6 weeks, 3 months, 6 months, and 12 months after the surgery. The following evaluations were accomplished at each postoperative visit: IKDC 2000 Subjective Knee Evaluation Form score; Knee Injury and Osteoarthritis Outcome Score (KOOS); IKDC 2000 Knee Examination Form; IKDC 2000 Current Health Assessment Form; 100-mm visual analog scale (VAS) at sitting, standing, and squatting; and the assessment of adverse events. A trained study nurse interviewed the patients and assisted the patients to complete the above assessments. Plain roentgenography, MRI, and second-look arthroscopy (only for patients who agreed to the additional procedure) were done at 12 months postoperatively. T1-weighted spoiled gradient echo (GRE), T2-weighted fast spin-echo and proton density images were conducted for MRI evaluation. The cartilage regeneration status was evaluated and graded as (1) fully regenerated, (2) partially regenerated, or (3) not regenerated. The Outerbridge classification was used to grade repaired tissue during arthroscopic examination. The evaluators of the MRI and arthroscopic video were blinded to the treatment.

### Rehabilitation protocol

Both patient groups underwent identical postoperative rehabilitation. For the first 6 weeks, patients used a knee brace with motion restricted to 0–90°. They were required to complete at least 500 passive knee flexion cycles daily and maintain partial weight bearing of up to 20 pounds (approximately the leg’s weight) using a heel–toe gait with two crutches. From week 6 to 8, patients gradually advanced to full weight bearing as tolerated.

### Study endpoints

The primary endpoint was the change in IKDC 2000 Subjective Knee Evaluation Form score from baseline. The secondary endpoints included the grade distribution for each domain of the IKDC 2000 Knee Examination Form and the amount of improvement evaluated by IKDC 2000 Current Health Assessment Form, KOOS, pain visual analog scales (VASs), MRI findings, and arthroscopic findings at 12 months.

### Statistical analysis

The patients were analyzed on an intention-to-treat (ITT) basis according to their randomization group. Missing data were accounted for by using the last observation carried forward method. For continuous variables, the number, mean, standard deviation, median, minimum, and maximum values were presented, and an analysis of variance (ANOVA), with treatment and study site as fixed effects, was performed to test the null hypothesis of prior-to-randomization comparability across treatment groups. For categorical variables, the numbers and percentages of subjects in each class were presented, and the Cochran–Mantel–Haenszel test adjusted for the study site was performed. The continuous efficacy outcomes were analyzed using an analysis of covariance (ANCOVA) with the baseline measure of that efficacy parameter as the covariate and effects of treatment and site as factors. Point estimates and 95% confidence intervals for the differences between the treatment groups were estimated. Summary statistics of the measured values, the percent changes, and the mean changes in the total IKDC-2000 Subjective Knee Evaluation Form score and other continuous efficacy outcomes were obtained for each group by observation time point, and their changes over time were also graphically presented.

## Results

**Fig. 2:**
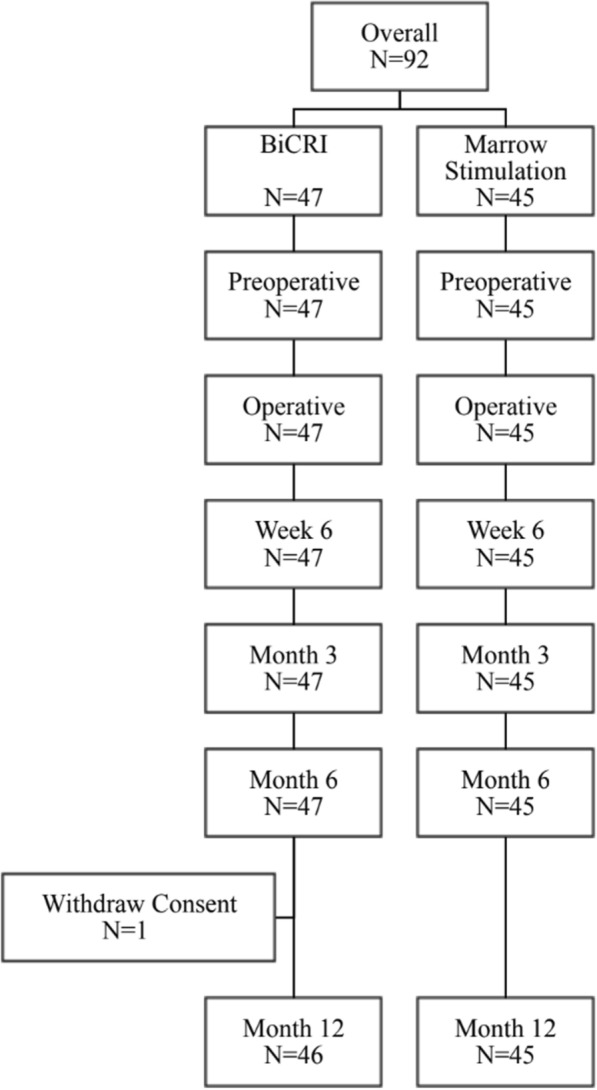
Flowchart of study population recruitment

During the study time period from November 2011 to March 2019, a total of 170 patients were assessed for eligibility, and 92 patients were enrolled. Forty-seven and 45 subjects were randomly assigned to the BiCRI group and microfracture group, respectively. All subjects were Asian or Pacific Islanders. One subject in the BiCRI group withdrew their consent after the postoperative visit at the 6th month (Fig. 2). The demographics and baseline characteristics are shown in Table 1. There was no significant difference in the characteristics between the two groups.

**Table 1:** Demographics and baseline characteristics of the participating patients

Characteristics	BiCRI (*n* = 47)	Microfracture (*n* = 45)	*P* value
Age (years)			
Mean (SD)	31.4(11.62)	30.9(11.23)	0.84
Height (cm)			
Mean (SD)	170.07 (6.96)	171.24 (8.63)	0.44
Weight (kg)			
Mean (SD)	74.02 (12.71)	73.02 (12.54)	0.72
Body mass index (kg/m^2^)			
Mean (SD)	25.59 (4.21)	24.82 (3.37)	0.33
Gender			
Male	37 (78.7%)	35 (77.8%)	0.94
Female	10 (21.3%)	10 (22.2%)	
Lesion size			
Lesion size ≤ 12.5 mm	32 (68.1%)	31 (68.9%)	0.90
Lesion size > 12.5 mm	15 (31.9%)	14 (31.1%)	
Smoking history			
Current smoker	9 (19.1%)	7 (15.6%)	0.96
Quit in the last 6 months	2 (4.3%)	2 (4.4%)	
Quit more than 6 months ago	3 (6.4%)	4 (8.9%)	
Has never smoked	33 (70.2%)	32 (71.1%)	
Education level			
Less than high school	4 (8.5%)	3 (6.7%)	0.85
Graduated from high school	12 (25.5%)	11 (24.4%)	
Some college education	8 (17.0%)	12 (26.7%)	
Graduated from college	18 (38.3%)	15 (33.3%)	
Postgraduate school or degree	5 (10.6%)	4 (8.9%)	
Activity level			
A high competitive sports person	7 (14.9%)	6 (13.3%)	0.85
Well trained and frequently participates in sports	14 (29.8%)	17 (37.8%)	
Ssometimes participates in sports	18 (38.3%)	15 (33.3%)	
Does not participate in sports	8 (17.0%)	7 (15.6%)	
IKDC-2000 Subjective Knee Evaluation Form			
Mean (SD)	60.11 (15.49)	59.64 (17.38)	0.87

*P* value: ANCOVA with treatment and site as covariates for continuous variables; Cochran–Mantel–Haenszel test adjusted for the study site for categorical variables

### Primary endpoint analysis

**Fig. 3:**
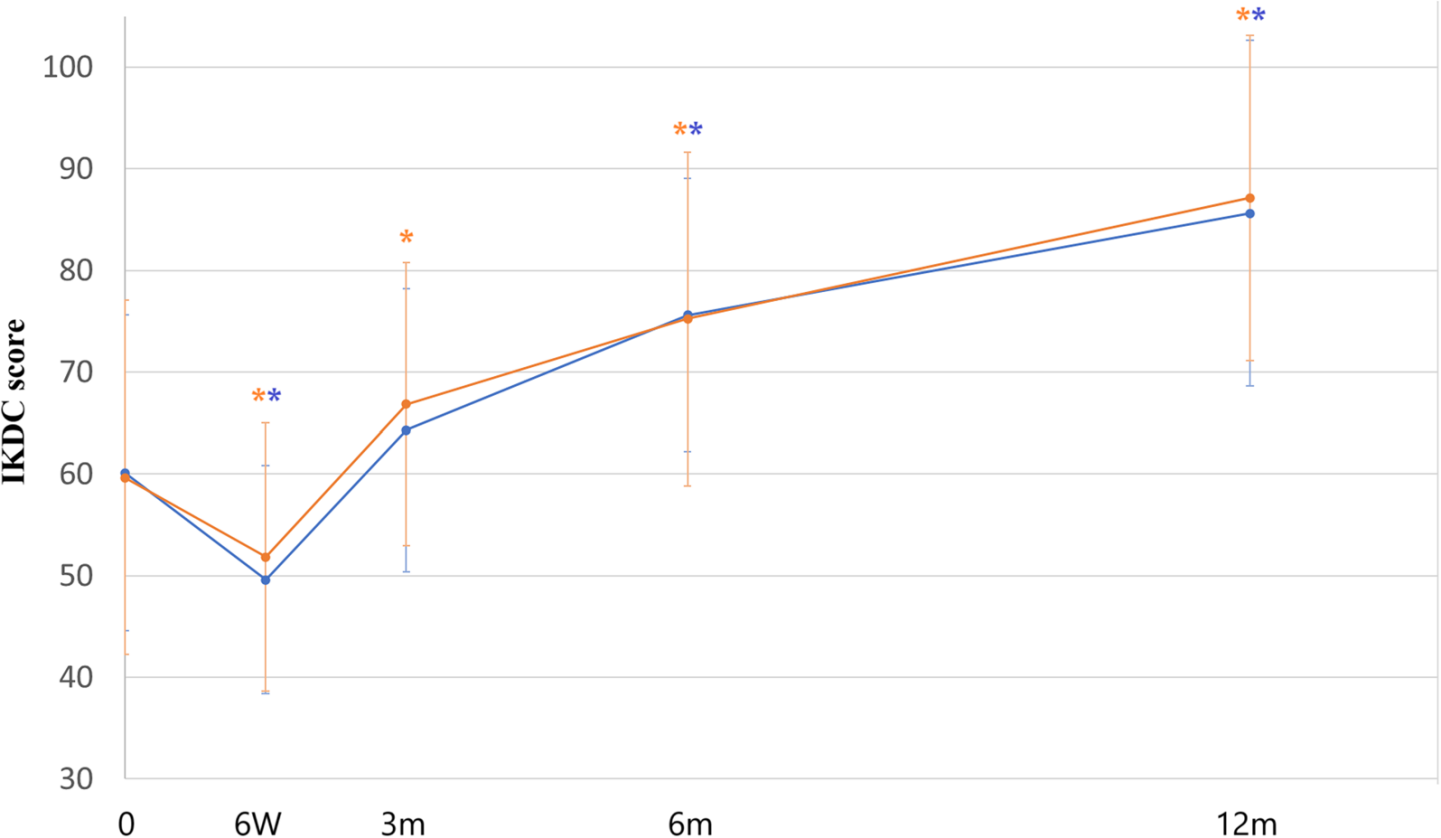
The IKDC score over time.* Blue line*: BiCRI group;* orange line*: microfracture group. The IKDC scores for both groups were comparable at all time points. * Significant improvement when compared to the baseline value

The IKDC scores at 6 weeks, 3 months, 6 months, and 12 months are shown in Table 2 and Fig. 3. At 12 months, the IKDC total scores were 85.61 ± 16.96 and 87.15 ± 15.98 for the BiCRI and microfracture arms, respectively. The change in the mean total score was 25.56 ± 18.48 points for the BiCRI arm and 27.51 ± 23.65 points for the microfracture arm. The lower limit of the two-sided 95% confidence interval (CI) for the score difference (BiCRI minus microfracture) between study treatments was − 6.95 points. This value is higher than the adopted non-inferiority margin of − 12 points, which indicates that BiCRI is non-inferior to microfracture surgery at 12 months.

**Table 2:** Change in the mean IKDC-2000 Subjective Knee Evaluation Form total score

	BiCRI (*n* = 47)	Microfracture (*n* = 45)	Treatment difference^c^ (BiCRI − microfracture)
Preoperative			
*N*	47	45	0.54 (3.370^d^)
Mean (SD)	60.11 (15.488)	59.64 (17.382)	
(Min, Max)	(27.6, 96.6)	(19.5, 97.7)	
*P* value			0.8741^a^
Week 6			
*N*	47	45	
Mean (SD)	49.60 (11.236)	51.83 (13.176)	
(Min, max)	(28.7, 72.4)	(21.8, 88.5)	
Mean change from baseline			
Mean (SD)	− 10.52 (16.657)	− 7.82 (17.510)	− 1.87 (2.371^d^)
95% CI	− 15.407 to − 5.625	− 13.077 to − 2.555	− 6.584 to 2.842
*P* value	< 0.0001^b^	0.0045^b^	0.4321^a^
Month 3			
*N*	47	45	
Mean (SD)	64.29 (13.912)	4.18 (18.173)	
(Min, max)	(35.6, 89.7)	(33.3, 100.0)	
Mean change from baseline			
Mean (SD)	4.18 (18.173)	7.20 (19.679)	− 1.96 (2.586^d^)
95% CI	− 1.154 to 9.518	1.291 to 13.115	− 7.096 to 3.184
*P* value	0.12^b^	0.02^b^	0.45^a^
Month 6			
*N*	47	45	
Mean (SD)	75.62 (13.357)	75.25 (16.358)	
(Min, max)	(43.7, 100.0)	(25.3, 100.0)	
Mean change from baseline			
Mean (SD)	15.51 (19.421)	15.61 (22.887)	0.79 (2.694^d^)
95% CI	9.803 to 21.207	8.731 to 22.483	−4.567 to 6.146
*P* value	< 0.0001^b^	< 0.0001^b^	0.77^a^
Month 12			
*N*	46	45	
Mean (SD)	85.61 (16.960)	87.15 (15.980)	
(Min, max)	(43.7, 100.0)	(35.6, 100.0)	
Mean change from baseline			
Mean (SD)	25.56 (18.476)	27.51 (23.651)	− 1.61 (2.684^d^)
95% CI	20.076 to 31.049	20.404 to 34.615	− 6.950 to 3.721
*P* value	< 0.0001^b^	< 0.0001^b^	0.55^a^
End of study			
*N*	47	45	
Mean (SD)	85.33 (16.885)	87.15 (15.980)	
(Min, max)	(43.7, 100.0)	(35.6, 100.0)	
Mean change from baseline			
Mean (SD)	25.21 (18.429)	27.51 (23.651)	− 1.56 (2.652^d^)
95% CI	19.803 to 30.625	20.404 to 34.615	− 6.835 to 3.708
*P* value	< 0.0001^b^	< 0.0001^b^	0.56^a^

^a^Inter-group* P* value from the ANCOVA model (outcome = treatment + site + error) for the preoperative visit.

Model for mean change value: outcome = treatment + site + baseline + error.* P* value is from testing the difference in treatment effect between study groups

^b^Intra-group* P* value from the paired* t*-test

^c^Least squares estimation

^d^For the treatment difference column, the mean (with the standard errer in parentheses) is shown

### Secondary endpoint analysis

**Fig. 4:**
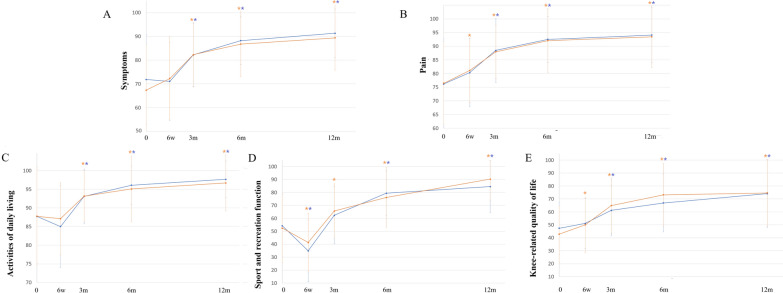
The KOOS scale over time.* Blue line*: BiCRI group; orange line: microfracture group. The KOOS results for both groups were comparable at all time points. * Significant improvement when compared to the baseline value. **A** Symptoms. **B** Pain. **C** Activities of daily living. **D** Sport and recreation function. **E** Knee-related quality of life

**Fig. 5:**
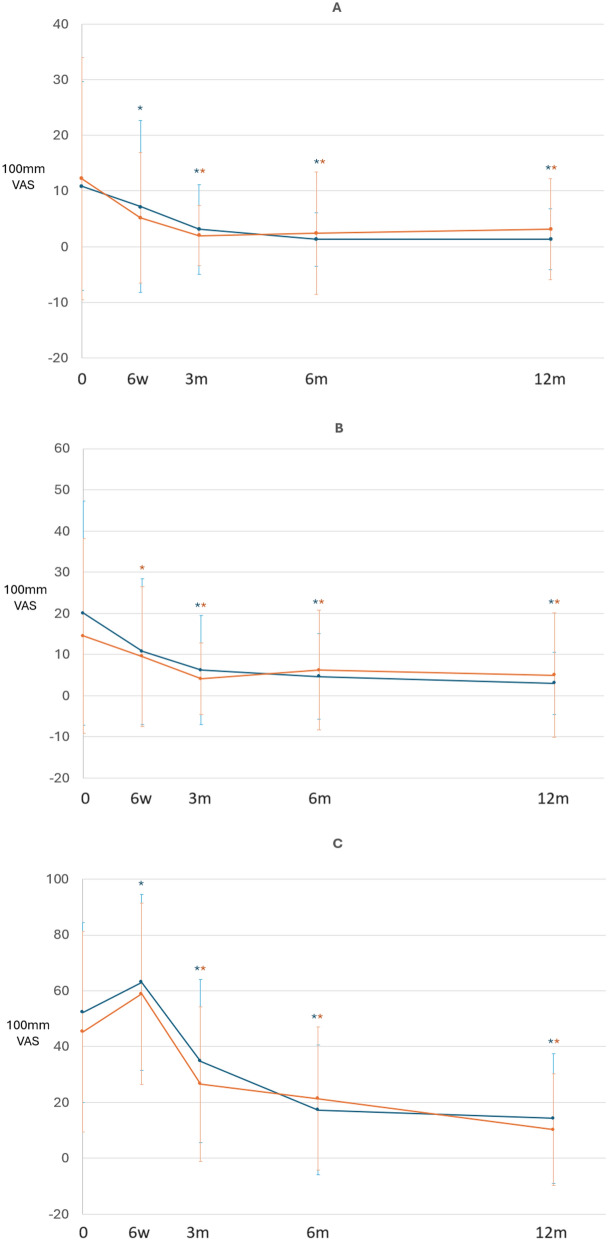
The pain VASs over time.* Blue line*: BiCRI group; orange line: microfracture group. **A** VAS when sitting. **B** VAS when standing. **C** VAS when squatting. The pain VAS results for both groups were comparable at all time points. * Significant improvement when compared to the baseline value

There was no significant difference between treatments in the grade distribution for each domain of the IKDC-2000 Knee Examination Form (the upper section of Table 3) and the amount of improvement evaluated by the IKDC-2000 Current Health Assessment Form (the lower section of Table 3), KOOS (Fig. 4), or the pain VASs (Fig. 5) at 12 months.

**Table 3:** Summary of the IKDC Knee Examination Form and IKDC-2000 Current Health Assessment Form results

IKDC Knee Examination Form			
Characteristics	BiCRI (*n* = 47)	Microfracture (*n* = 45)	*P* value
Effusion			
Preoperative (*N*)	47	45	0.14
Normal	34 (72.3%)	40 (88.9%)	
Nearly normal	9 (19.1%)	4 (8.9%)	
Abnormal	4 (8.5%)	1 (2.2%)	
Severely abnormal	0	0	
Month 12 (*N*)	46	45	
Normal	42 (91.3%)	44 (97.8%)	0.16
Nearly normal	4 (8.7%)	1 (2.2%)	
Abnormal	0	0	
Severely abnormal	0	0	
Passive motion deficit			
Preoperative (N)	47	45	0.35
Normal	40 (85.1%)	41 (91.1%)	
Nearly normal	5 (10.6%)	3 (6.7%)	
Abnormal	0	1 (2.2%)	
Severely abnormal	2 (4.3%)	0	
Month 12 (*N*)	46	45	
Normal	46 (100.0%)	45 (100.0%)	-
Nearly normal	0	0	
Abnormal	0	0	
Severely abnormal	0	0	
Ligament examination			
Preoperative (*N*)	47	45	0.02
Normal	32 (68.1%)	31 (68.9%)	
Nearly normal	5 (10.6%)	0	
Abnormal	10 (21.3%)	10 (22.2%)	
Severely abnormal	0	4 (8.9%)	
Month 12 (*N*)	46	45	
Normal	44 (95.7%)	44 (97.8%)	0.60
Nearly normal	2 (4.3%)	1 (2.2%)	
Abnormal	0	0	
Severely abnormal	0	0	
Compartment findings			
Preoperative (*N*)	47	45	0.89
Normal	33 (70.2%)	33 (73.3%)	
Nearly normal	10 (21.3%)	8 (17.8%)	
Abnormal	4 (8.5%)	4 (8.9%)	
Severely abnormal	0	0	
Month 12 (*N*)	46	45	
Normal	42 (91.3%)	39 (86.7%)	0.51
Nearly normal	3 (6.5%)	5 (11.1%)	
Abnormal	0	5 (11.1%)	
Severely abnormal	1 (2.2%)	0	
Harvest site pathology			
Preoperative (*N*)	47	45	0.78
Normal	37 (78.7%)	36 (80.0%)	
Nearly normal	6 (12.8%)	7 (15.6%)	
Abnormal	4 (8.5%)	2 (4.4%)	
Severely abnormal	0	0	
Month 12 (*N*)	46	45	
Normal	45 (97.8%)	43 (95.6%)	0.67
Nearly normal	1 (2.2%)	2 (4.4%)	
Abnormal	0	0	
Severely abnormal	0	0	
X-ray findings			
Preoperative (*N*)	47	45	0.03
Normal	42 (89.4%)	33 (73.3%)	
Nearly normal	4 (8.5%)	12 (26.7%)	
Abnormal	1 (2.1%)	0	
Severely abnormal	0	0	
Month 12 (*N*)	46	45	
Normal	40 (87.0%)	39 (86.7%)	0.54
Nearly normal	5 (10.9%)	6 (13.3%)	
Abnormal	1 (2.2%)	0	
Severely abnormal	0	0	
Functional test			
Preoperative (*N*)	47	45	0.33
Normal	11 (23.4%)	16 (35.6%)	
Nearly normal	11 (23.4%)	11 (24.4%)	
Abnormal	9 (19.1%)	10 (22.2%)	
Severely abnormal	16 (34.0%)	8 (17.8%)	
Month 12 (*N*)	46	45	
Normal	31 (67.4%)	32 (71.1%)	0.72
Nearly normal	11 (23.9%)	9 (20.0%)	
Abnormal	4 (8.7%)	3 (6.7%)	
Severely abnormal	0	1 (2.2%)	
Final evaluation			
Preoperative (*N*)	47	45	0.05
Normal	19 (40.4%)	26 (57.8%)	
Nearly normal	13 (27.7%)	3 (6.7%)	
Abnormal	13 (27.7%)	12 (26.7%)	
Severely abnormal	2 (4.3%)	4 (8.9%)	
Month 12 (*N*)	46	45	
Normal	40 (87.0%)	43 (95.6%)	0.15
Nearly normal	6 (13.0%)	2 (4.4%)	
Abnormal	0	0	
Severely abnormal	0	0	

IKDC 2000 Current Health Assessment Form			
	BiCRI (*n* = 47)	Microfracture (*n* = 45)	*P* value^a^
Physical functioning			
Preoperative (N)	47	45	
Mean (SD)	65.53 (24.896)	68.44 (22.203)	0.53
Month 12 (*N*)	46	45	
Mean (SD)	94.02 (10.307)	90.00 (22.335)	
Mean change (SD)	28.70 (24.414)	21.56 (32.471)	0.27
*P* value^b^	< 0.0001	< 0.0001	
Role—physical			
Preoperative (*N*)	47	45	
Mean (SD)	25.53 (38.831)	25.53 (38.831)	0.78
Month 12 (*N*)	46	45	
Mean (SD)	85.87 (28.212)	85.00 (32.596)	
Mean change (SD)	59.78 (45.789)	58.33 (51.676)	0.82
*P* value^b^	< 0.0001	< 0.0001	
Role—emotional			
Preoperative (*N*)	47	45	
Mean (SD)	53.19 (45.926)	46.67 (44.608)	0.62
Month 12 (*N*)	46	45	
Mean (SD)	94.20 (18.992)	91.85 (23.736)	
Mean change (SD)	40.58 (49.647)	45.19 (51.813)	0.65
*P* value^b^	< 0.0001	< 0.0001	
Vitality			
Preoperative (*N*)	47	45	
Mean (SD)	80.85 (19.981)	76.56 (24.745)	0.14
Month 12 (*N*)	46	45	
Mean (SD)	86.52 (18.008)	83.67 (19.926)	
Mean change (SD)	5.43 (15.593)	7.11 (19.409)	0.57
*P* value^b^	0.02	0.02	
Mental health			
Preoperative (*N*)	47	45	
Mean (SD)	83.51 (18.764)	79.33 (19.206)	0.14
Month 12 (*N*)	46	45	
Mean (SD)	88.04 (18.089)	88.33 (16.307)	
Mean change (SD)	4.67 (18.300)	9.00 (17.825)	0.06
*P* value^b^	0.09	0.0015	
Social functioning			
Preoperative (*N*)	47	45	
Mean (SD)	72.07 (24.618)	71.94 (22.949)	0.86
Month 12 (*N*)	46	45	
Mean (SD)	94.02 (13.107)	94.44 (12.657)	
Mean change (SD)	21.74 (23.634)	22.50 (26.328)	0.68
*P* value^b^	< 0.0001	< 0.0001	
Bodily pain			
Preoperative (*N*)	47	45	
Mean (SD)	57.21 (20.478)	58.67 (20.672)	0.65
Month 12 (*N*)	46	45	
Mean (SD)	88.02 (18.249)	89.49 (19.151)	
Mean change (SD)	30.91 (19.530)	30.82 (26.262)	0.67
*P* value^b^	< 0.0001	< 0.0001	
General health			
Preoperative (*N*)	47	45	
Mean (SD)	76.87 (18.986)	74.42 (23.598)	0.35
Month 12 (*N*)	46	45	
Mean (SD)	85.78 (18.657)	84.71 (21.114)	
Mean change (SD)	8.70 (15.768)	10.29 (19.518)	0.87
*P* value^b^	0.0005	0.0010	

*P* value: Cochran–Mantel–Haenszel test adjusted for the study site for the IKDC Knee Examination Form

*P* value: ^a^inter-group* P* value from ANCOVA; ^b^intra-group* P* value from a paired *t*-test for the IKDC-2000 Current Health Assessment Form

**Fig. 6:**
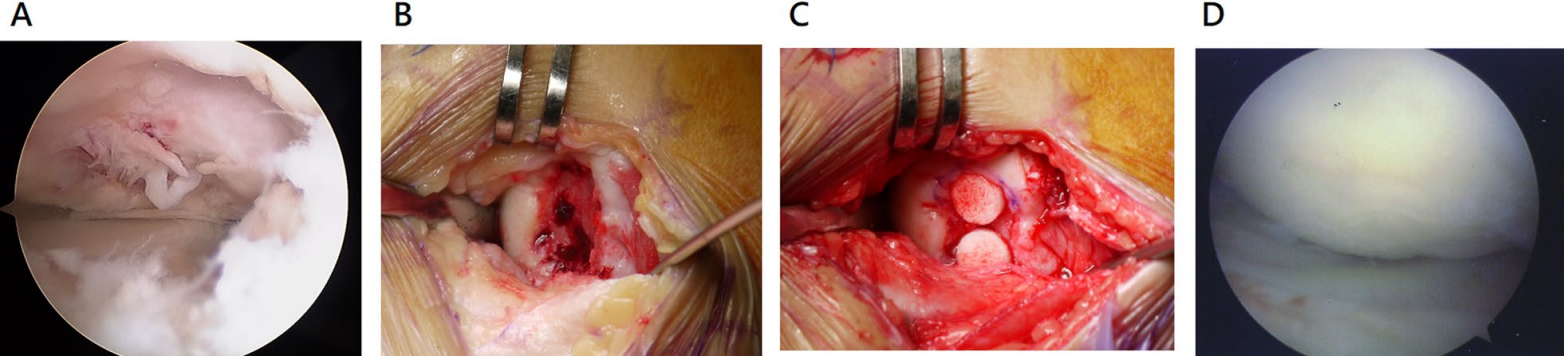
Representative photo of fully regenerated cartilage after BiCRI. **A** Arthroscopic photo before BiCRI. **B** After debridement of the defect. **C** After BiCRI implantation. **D** Arthroscopy at 12 months showed fully regenerated cartilage

T2-weighted fast spin-echo MRI at 12 months showed that 97.7% of the patients in the BiCRI arm (44/45) and 86.7% of the patients in the microfracture arm (39/45) had their defects repaired with fully regenerated or partially regenerated tissue. Similarly, proton density MRI showed that 97.7% of the BiCRI patients (44/45) and 88.9% of the microfracture patients (40/45) had their defects repaired with fully regenerated or partially regenerated tissue. The MRI findings are listed in the upper section of Table 4. Twenty-six patients in the BiCRI arm and 22 patients in the microfracture arm underwent arthroscopic examination at 12 months. The arthroscopic findings for the repaired defects are shown in the lower section of Table 4. A representative arthroscopic photograph of fully regenerated cartilage is presented in Fig. 6.

**Table 4:** Summary of the cartilage regeneration status

MRI evaluation			
	BiCRI (*n* = 47)	Microfracture (*n* = 45)	*P* value
T2-weighted fast spin-echo image (12th month)			
*N*	45	45	
Not evaluated	0	1 (2.2%)	0.20
Fully regenerated	20 (44.4%)	14 (31.1%)	
Partially regenerated	24 (53.3%)	25 (55.6%)	
Not regenerated	1 (2.2%)	5 (11.1%)	
Proton density image (12th month)			
*N*	45	45	
Not evaluated	0	0	0.14
Fully regenerated	20 (44.4%)	14 (31.1%)	
Partially regenerated	24 (53.3%)	26 (57.8%)	
Not regenerated	1 (2.2%)	5 (11.1%)	

Arthroscopic evaluation		
	BiCRI (*n* = 26)	Microfracture (*n* = 22)
Grade 0	3	0
Grade I	9	11
Grade II	12	7
Grade III	0	O
Grade IV	1	2
Not evaluable	1	2

*P* value: Cochran-Mantel–Haenszel test adjusted for the study site

Grade 0: normal cartilage

Grade I: cartilage with softening and swelling

Grade II: a partial-thickness defect with fibrillation or fissures on the surface that did not reach subchondral bone or exceed 1.5 cm in diameter

Grade III: fissuring to the level of the subchondral bone in an area with a diameter of more than 1.5 cm

Grade IV: exposed subchondral bone

### Safety evaluation

There were no device-related adverse events reported in this study, and no deaths occurred. Treatment-related adverse events are shown in Table 5. Procedural pain was the most commonly reported procedure-related adverse event, with rates of 78.7% for BiCRI and 77.8% for marrow stimulation.

**Table 5:** Treatment-related adverse events

	BiCRI (*n* = 47)	Microfracture (*n* = 45)	*P* value
Procedural pain	37 (78.7%)	35 (77.8%)	1.000
Swelling	24 (51.1%)	15 (33.3%)	0.096
Joint effusion	9 (19.1%)	8 (17.8%)	1.000
Arthralgia	11 (23.4%)	15 (33.3%)	0.357
Dizziness	2 (4.3%)	1 (2.2%)	1.000

In the microfracture group, one patient had seven serious adverse events (SAEs), including uterine leiomyoma, hydronephrosis, hydroureter, acute pyelonephritis, renal impairment, urinary tract infection, and endometriosis. None of the SAEs in this patient were related to the procedure.

## Discussion

In this study, we demonstrated that BiCRI is non-inferior to microfracture surgery for treating chondral or osteochondral defects in the knee, specifically in terms of the subjective improvement at 1 year. Outcome measures reflecting patient functioning across various health domains play a crucial role in evaluating the effectiveness of cartilage repair studies and monitoring individual patient progress. The FDA Advisory Panel has emphasized the inclusion of both pain and function measurements in the primary endpoint for cartilage repair therapies. In the present study, we designated the IKDC 2000 Subjective Knee Evaluation Form (IKDC SKF) as our primary efficacy endpoint. The IKDC SKF has been validated as a suitable knee-specific instrument for assessing symptoms, daily function, and the level of symptom-free sports activity in patients undergoing articular cartilage surgery [28]. We deliberately chose a small effect size to define the non-inferiority margin. Furthermore, the loss-to-follow-up rate was satisfactorily low in both arms, bolstering our confidence that the subjective improvement, as indicated by the increased IKDC scores in the BiCRI arm, was at least as good as that in the microfracture arm at 12 months. In comparison to the preoperative status, a significant increase in IKDC scores occurred more gradually in the BiCRI arm (6 months versus 3 months). We speculate that the more intricate biological processes involved in BiCRI, such as chondrocyte migration, proliferation, and subchondral integration, necessitated a longer regeneration time. However, a stable improvement in knee function could be anticipated after 12 months [23].

Microfracture is the most commonly employed reparative technique for addressing articular cartilage defects in the knee [6, 29]. It has served as the primary treatment option due to its simplicity and cost-effectiveness [6, 29–31]. In the literature, the rate of short-term clinical improvement after microfracture consistently ranges from 75 to 100% [29]. Consequently, it is frequently utilized as a standard for comparing other reparative or regenerative procedures [11, 12, 29, 32–34]. Functional outcomes, as assessed by IKDC scores, Lysholm scores, or KOOS, have been found to be comparable between patients treated with ACI and microfracture within a 5-year timeframe [12, 34]. Despite questions about the durability of the initial improvement after microfracture [35], it remains a suitable control treatment for comparing short-term clinical functional outcomes [12, 32]. In the current study, we conducted a comprehensive evaluation of clinical outcomes using the IKDC 2000 Knee Examination Form, IKDC 2000 Current Health Assessment Form, KOOS, and pain VASs as secondary endpoints. The results were comparable between patients treated with BiCRI and microfracture at 12 months. The evaluations encompassed almost every aspect of knee function, including physical activities, knee-related quality of life, social functioning, mental health, X-ray findings, and pain in different positions. These results indicate that BiCRI is a suitable alternative treatment for chondral or osteochondral defects.

Based on the MRI findings, fully or partially regenerated cartilage was observed in more than 95% of the BiCRI patients and 85% of the microfracture patients with defects. Although there was no significant difference in cartilage regeneration status between both groups, the MRI evaluation could not definitively determine whether the regenerated cartilage was fibrocartilage or hyaline-like cartilage. It is well known that microfracture can only induce fibrocartilage formation [36], which is mechanically weaker than hyaline cartilage and lacks the intrinsic biochemical and viscoelastic properties of normal articular cartilage. Consequently, it is associated with poorer mid- to long-term outcomes [37]. In contrast, our previous studies [22, 23] demonstrated that the tissue regenerated after BiCRI implantation is hyaline in nature, as confirmed by positive staining with Alcian blue and immunohistological staining for collagen type II. Promising mid-term outcomes for BiCRI have also been reported previously [23]. Moreover, MRI has inherent limitations when precisely assessing defects due to its limited number of slices, and it offers only restricted insight into cartilage quality and composition. Arthroscopic findings further validated that BiCRI was not inferior to microfracture surgery. Over 80% of the patients in both groups exhibited low-grade (≤ grade 2) cartilage, with a higher proportion of grade 0 cartilage observed in the BiCRI group. These findings suggest that BiCRI may be a more effective treatment option. An example of grade 0 regenerated cartilage is shown in Fig. 6.

The safety findings of this study affirm the short-term safety of the biphasic construct. The rates of adverse events were comparable between the BiCRI and microfracture arms. The adverse events consisted of common postoperative symptoms, including procedural pain, swelling, arthralgia, and joint effusion, all of which were temporary and resolved within months. Therefore, we conclude that there are no safety concerns regarding the biphasic construct. These safety findings align with previous short- to mid-term reports in a clinical feasibility study [23].

This study has several limitations. Firstly, the trial was designed as a non-inferiority study, limiting our conclusions to confirming that BiCRI is non-inferior to microfracture. Given the greater complexity and higher cost of the BiCRI procedure compared to microfracture, further trials with a superiority design are necessary to establish its cost-effectiveness. Secondly, blinding the patients was challenging as they could potentially discern their treatment through intentional image assessment without notifying the researchers. Consequently, the risk of bias due to a placebo effect cannot be entirely ruled out. Additionally, the current presentation only permits the comparison of short-term outcomes. Compared to other similar studies, the follow-up period is relatively short [16, 17]. As the mid-term outcomes from a prior clinical feasibility study were promising [23], a more extended follow-up is warranted to demonstrate the durability of the clinical efficacy.

## Conclusion

Based on IKDC 2000 Subjective Knee Evaluation Form scores, BiCRI proved non-inferior to microfracture at 12 months. Short-term functional outcomes were comparable to those of microfracture, while arthroscopic findings showed more complete cartilage regeneration in the BiCRI group. Consequently, BiCRI emerges as a viable alternative for treating chondral or osteochondral defects.

## Data Availability

The datasets used and/or analyzed during the current study are available from the corresponding author on reasonable request.

## Abbreviations

ACI: Autologous cartilage implantation
ANOVA: Analysis of variance
BiCRI: Biphasic cartilage repair implant
BMI: Body mass index
DSMB: Data safety monitoring board
IKDC SKF: International Knee Documentation Committee 2000 Subjective Knee Evaluation Form
ITT: Intention to treat
KOOS: Knee Injury and Osteoarthritis Outcome Score
MACI: Matrix-associated autologous chondrocyte implantation
OA: Osteoarthritis
PLGA: Polylactic-*co*-glycolic acid
TCP: Tricalcium phosphate
VAS: Visual analog scale

## References

[1] Arøen A, Løken S, Heir S et al (2004) Articular cartilage lesions in 993 consecutive knee arthroscopies. Am J Sports Med 32:211–215 14754746 10.1177/0363546503259345

[2] Heir S, Nerhus TK, Røtterud JH et al (2010) Focal cartilage defects in the knee impair quality of life as much as severe osteoarthritis: a comparison of knee injury and osteoarthritis outcome score in 4 patient categories scheduled for knee surgery. Am J Sports Med 38:231–237 20042546 10.1177/0363546509352157

[3] Strauss EJ, Goodrich LR, Chen CT, Hidaka C, Nixon AJ (2005) Biochemical and biomechanical properties of lesion and adjacent articular cartilage after chondral defect repair in an equine model. Am J Sports Med 33:1647–1653 16093540 10.1177/0363546505275487

[4] Tuan RS, Chen AF, Klatt BA (2013) Cartilage regeneration. J Am Acad Orthop Surg 21:303–311 23637149 10.5435/JAAOS-21-05-303 PMC4886741

[5] Shapiro F, Koide S, Glimcher MJ (1993) Cell origin and differentiation in the repair of full-thickness defects of articular cartilage. J Bone Joint Surg Am 75:532–553 8478382 10.2106/00004623-199304000-00009

[6] McCormick F, Harris JD, Abrams GD et al (2014) Trends in the surgical treatment of articular cartilage lesions in the United States: an analysis of a large private-payer database over a period of 8 years. Arthroscopy 30:222–226 24485115 10.1016/j.arthro.2013.11.001

[7] Gomoll AH, Minas T (2014) The quality of healing: articular cartilage. Wound Repair Regen 22(Suppl 1):30–38 24813362 10.1111/wrr.12166

[8] Peterson L, Vasiliadis HS, Brittberg M, Lindahl A (2010) Autologous chondrocyte implantation: a long-term follow-up. Am J Sports Med 38:1117–1124 20181804 10.1177/0363546509357915

[9] Mundi R, Bedi A, Chow L et al (2016) Cartilage restoration of the knee: a systematic review and meta-analysis of level 1 studies. Am J Sports Med 44:1888–1895 26138733 10.1177/0363546515589167

[10] Knutsen G, Engebretsen L, Ludvigsen TC et al (2004) Autologous chondrocyte implantation compared with microfracture in the knee. A randomized trial. J Bone Joint Surg Am 86:455–464 14996869 10.2106/00004623-200403000-00001

[11] Knutsen G, Drogset JO, Engebretsen L et al (2016) A randomized multicenter trial comparing autologous chondrocyte implantation with microfracture: long-term follow-up at 14 to 15 years. J Bone Joint Surg Am 98:1332–1339 27535435 10.2106/JBJS.15.01208

[12] Van Assche D, Staes F, Van Caspel D et al (2010) Autologous chondrocyte implantation versus microfracture for knee cartilage injury: a prospective randomized trial, with 2-year follow-up. Knee Surg Sports Traumatol Arthrosc 18:486–495 19820916 10.1007/s00167-009-0955-1

[13] Brittberg M, Lindahl A, Nilsson A, Ohlsson C, Isaksson O, Peterson L (1994) Treatment of deep cartilage defects in the knee with autologous chondrocyte transplantation. N Engl J Med 331:889–895 8078550 10.1056/NEJM199410063311401

[14] Bartlett W, Skinner JA, Gooding CR et al (2005) Autologous chondrocyte implantation versus matrix-induced autologous chondrocyte implantation for osteochondral defects of the knee: a prospective, randomised study. J Bone Joint Surg Br 87:640–645 15855365 10.1302/0301-620X.87B5.15905

[15] Filardo G, Kon E, Roffi A, Di Martino A, Marcacci M (2013) Scaffold-based repair for cartilage healing: a systematic review and technical note. Arthroscopy 29:174–186 23159494 10.1016/j.arthro.2012.05.891

[16] Uchio Y, Kuroda R, Niki Y, Sugawara K, Ishibashi Y (2024) Effectiveness and safety of matrix-associated autologous chondrocyte implantation for the treatment of articular cartilage defects: a real-world data analysis in Japan. Am J Sports Med. 10.1177/03635465241282671 39397727 10.1177/03635465241282671

[17] Gaissmaier C, Angele P, Spiro RC, Köhler A, Kirner A, Niemeyer P (2024) Hydrogel-based matrix-associated autologous chondrocyte implantation shows greater substantial clinical benefit at 24 months follow-up than microfracture: a propensity score matched-pair analysis. Cartilage. 10.1177/19476035241235928 38501741 10.1177/19476035241235928 PMC11569661

[18] Hoburg A, Niemeyer P, Laute V et al (2023) Sustained superiority in KOOS subscores after matrix-associated chondrocyte implantation using spheroids compared to microfracture. Knee Surg Sports Traumatol Arthrosc 31:2482–2493 36269383 10.1007/s00167-022-07194-x

[19] Chaipinyo K, Oakes BW, Van Damme MP (2004) The use of debrided human articular cartilage for autologous chondrocyte implantation: maintenance of chondrocyte differentiation and proliferation in type I collagen gels. J Orthop Res 22:446–455 15013108 10.1016/j.orthres.2003.07.001

[20] Chiang H, Liao CJ, Wang YH et al (2010) Comparison of articular cartilage repair by autologous chondrocytes with and without in vitro cultivation. Tissue Eng Part C Methods 16:291–300 20187869 10.1089/ten.tec.2009.0298

[21] Lu Y, Dhanaraj S, Wang Z et al (2006) Minced cartilage without cell culture serves as an effective intraoperative cell source for cartilage repair. J Orthop Res 24:1261–1270 16652342 10.1002/jor.20135

[22] Chiang H, Liao CJ, Hsieh CH, Shen CY, Huang YY, Jiang CC (2013) Clinical feasibility of a novel biphasic osteochondral composite for matrix-associated autologous chondrocyte implantation. Osteoarthr Cartil 21:589–598 10.1016/j.joca.2013.01.004 23333470

[23] Tseng TH, Jiang CC, Lan HH, Chen CN, Chiang H (2020) The five year outcome of a clinical feasibility study using a biphasic construct with minced autologous cartilage to repair osteochondral defects in the knee. Int Orthop 44:1745–1754 32367232 10.1007/s00264-020-04569-y

[24] Kon E, Gobbi A, Filardo G, Delcogliano M, Zaffagnini S, Marcacci M (2009) Arthroscopic second-generation autologous chondrocyte implantation compared with microfracture for chondral lesions of the knee: prospective nonrandomized study at 5 years. Am J Sports Med 37:33–41 19059899 10.1177/0363546508323256

[25] Liao CJ, Chen CF, Chen JH, Chiang SF, Lin YJ, Chang KY (2002) Fabrication of porous biodegradable polymer scaffolds using a solvent merging/particulate leaching method. J Biomed Mater Res 59:676–681 11774329 10.1002/jbm.10030

[26] Liao CJ, Lin YJ, Chiang H, Chiang SF, Wang YH, Jiang CC (2007) Injecting partially digested cartilage fragments into a biphasic scaffold to generate osteochondral composites in a nude mice model. J Biomed Mater Res A 81:567–577 17177287 10.1002/jbm.a.31035

[27] Chiang H, Kuo TF, Tsai CC et al (2005) Repair of porcine articular cartilage defect with autologous chondrocyte transplantation. J Orthop Res 23:584–593 15885479 10.1016/j.orthres.2004.11.003

[28] Greco NJ, Anderson AF, Mann BJ et al (2010) Responsiveness of the International Knee Documentation Committee Subjective Knee Form in comparison to the Western Ontario and McMaster Universities Osteoarthritis Index, modified Cincinnati Knee Rating System, and Short Form 36 in patients with focal articular cartilage defects. Am J Sports Med 38:891–902 20044494 10.1177/0363546509354163

[29] Mithoefer K, McAdams T, Williams RJ, Kreuz PC, Mandelbaum BR (2009) Clinical efficacy of the microfracture technique for articular cartilage repair in the knee: an evidence-based systematic analysis. Am J Sports Med 37:2053–2063 19251676 10.1177/0363546508328414

[30] Aae TF, Randsborg PH, Lurås H, Årøen A, Lian ØB (2018) Microfracture is more cost-effective than autologous chondrocyte implantation: a review of level 1 and level 2 studies with 5 year follow-up. Knee Surg Sports Traumatol Arthrosc 26:1044–1052 29128878 10.1007/s00167-017-4802-5 PMC5876257

[31] Mistry H, Connock M, Pink J et al (2017) Autologous chondrocyte implantation in the knee: systematic review and economic evaluation. Health Technol Assess 21:1–294 28244303 10.3310/hta21060 PMC5346885

[32] Yoon KH, Yoo JD, Choi CH et al (2021) Costal chondrocyte-derived pellet-type autologous chondrocyte implantation versus microfracture for repair of articular cartilage defects: a prospective randomized trial. Cartilage 13:1092s–1104s 32476445 10.1177/1947603520921448 PMC8808917

[33] Na Y, Shi Y, Liu W et al (2019) Is implantation of autologous chondrocytes superior to microfracture for articular-cartilage defects of the knee? A systematic review of 5-year follow-up data. Int J Surg 68:56–62 31220632 10.1016/j.ijsu.2019.06.007

[34] Gou GH, Tseng FJ, Wang SH et al (2020) Autologous chondrocyte implantation versus microfracture in the knee: a meta-analysis and systematic review. Arthroscopy 36:289–303 31708355 10.1016/j.arthro.2019.06.033

[35] Kreuz PC, Steinwachs MR, Erggelet C et al (2006) Results after microfracture of full-thickness chondral defects in different compartments in the knee. Osteoarthr Cartil 14:1119–1125 10.1016/j.joca.2006.05.003 16815714

[36] Song SJ, Park CH (2019) Microfracture for cartilage repair in the knee: current concepts and limitations of systematic reviews. Ann Transl Med 7:S108 31576315 10.21037/atm.2019.05.11 PMC6685863

[37] Frank RM, Cotter EJ, Nassar I, Cole B (2017) Failure of bone marrow stimulation techniques. Sports Med Arthrosc Rev 25:2–9 28045867 10.1097/JSA.0000000000000134

